# Seven Factors Affecting Medical Undergraduate Students’ Performance in Academics: A Study Using Ron Fry Questionnaire in Eastern India

**DOI:** 10.30476/jamp.2020.86444.1239

**Published:** 2020-10

**Authors:** ABHISHEK DAS, SHUVRO BHATTACHARYA, ARANI CHAKRABORTY

**Affiliations:** 1 Upgraded Department of Forensic and State Medicine, Medical College Kolkata, 88 College Street, Pin-700073, West Bengal, India; 2 Medical College Kolkata, 88 College Street, Pin-700073, West Bengal, India

**Keywords:** Education, Medical, Undergraduate, Surveys and questionnaires, Anxiety, Attention

## Abstract

**Introduction:**

Undergraduate medical students are adult learners facing various assessments through examination during academic course, but the result is unsatisfactory. This study aimed at identifying the gaps in academics and finding out areas for improvement among the identified factors affecting the students’ performance.

**Methods:**

A questionnaire survey prospective, cross-sectional, observational, questionnaire- based study was conducted among the 3rd semester undergraduate students (N=215) in a Government Medical College of Eastern India by census method using predesigned, pretested, validated tool designed by Ron Fry. A total of 200 students participated in this study. A closed questionnaire containing 28 questions with dichotomous options was distributed. Data collected were tabulated in MS Excel spreadsheet and evaluated according to the proposed guidelines on seven factors: concentration, comprehension, test anxiety, organization, research aptitude, computer skill, and taking notes. Statistical significance of the data distribution among different groups was estimated using Chi-square tests in MS Excel 2010 software, and p<0.05 was considered as significant.

**Results:**

Among 200 participants (response rate=93%), 196 were (Male=142, Female= 54) accepted; the respondats’ age range was 18-22 years. Of them, 48 students obtained honours marks (≥75% in any subject). 137 (69.9%) students had lacunae in any of the above-mentioned domains, comprehension (97.9%) being the highest. The major determining factors were test anxiety along with note taking and concentration. The differences between the males and females regarding concentration (p=0.008) and note taking (p=0.009) were statistically significant. Test anxiety was the differentiating factor (p=0.013) between honours and non-honours candidates.

**Conclusion:**

Researches worldwide have identified extrinsic, intrinsic, personal, and miscellaneous factors affecting the students’ performances. This is a multifaceted issue which can be managed individually. Few of the most important determinants were dealt with in this study. To perform well, every student should understand what to learn, what to remember, and how to represent in examinations. This study will help the education authorities to guide the students and implement Competency Based Medical Education (CBME) based on Attitude, Ethics, and Communication (AETCOM) module in India.

## Introduction

Learning is an individual’s way of perceiving, processing, and retaining new information which ends with successful recall. Its outcome depends on many factors like age, sex, intelligence quotient (IQ), culture and ethnicity, mentality and attitude, preparedness, associated psychological conditions, and genetic factors of the learner ( 1 ). During medical academic course, learning is assessed by examination which is an integral part of any educational system, but it is miserable and stressful as well as results make no one happy. Many factors, personal, familial, internal, or external, have negative roles in achieving a favourable result. It’s important to identify the areas of lacunae as stress management or the coping strategy is not known by all ( 2 ). The researchers all over the world, through years, have been interested in finding such influencing factors and their interplay. Primarily, all university students are adult learners and their learning and performance are guided by seven principles of ‘andragogy’: providing safe and comfortable learning climate, getting involved in mutual planning, diagnosing one’s own needs to trigger internal motivation, formulating one’s own objectives to get more control on their learning, identifying and using the resources to achieve one’s own objectives, supporting learning plans and self-evaluation to develop skills of critical reflection ( 3 , 4 ). Still, many areas of improvement remain unnoticed and are overlooked. Ron Fry mentioned arguably the most important seven issues in his work on students and learning psychology and incorporated them in a questionnaire designed by him. These include concentration, comprehension, text anxiety, organisation skill, learning ability, computer skill, and note taking in this study ( 5 ). To the best of our knowledge, this study is the first of its kind to be applied for the medical undergraduate students. The objectives of this study were to find the area for improvement among these identified factors affecting medical undergraduate students’ performance at the university level and identify the gaps in academics between different groups of undergraduate medical students.

## Methods

The is a cross-sectional, prospective, observational questionnaire study done in a tertiary care government medical college in eastern India over a period of two months among all 3rd semester undergraduate medical students. Students of this year have been chosen as they have just attended for the first time in the University examination. Census method was used including all students of the mentioned batch. All the students who were present in class during the days of data collection, irrespective of their gender and age, were included in the study. Only the questionnaires filled out incompletely were excluded from this study. Predesigned, pretested, and validated questionnaire formulated by Ron Fry was used as the study tool ( 5 ). Before distribution of the questionnaire, proper informed consent for voluntary participation was obtained from each student. The questionnaires were then distributed after a lecture session in a single page printed format and then collected after the due time. The study was made anonymous by only receiving information about sex and age of the students, but no identifying information such as name was inquired. The collected data were tabulated in MS Excel 2010 spreadsheet and scored to find out the influencing factors. For each student, the responses were recorded according to the proposed guideline mentioned ( 5 ). The students were then divided into two groups according to sex and their secured distinction (Honours marks as defined for Indian medical Graduates, i.e. securing 75% or more marks in a particular subject) marks. The groups were compared using appropriate statistical tests using MS Excel 2010 software.

Predesigned, pretested, and validated questionnaire formulated by Ron Fry is a closed questionnaire comprising of 28 descriptive statements with dichotomous options (Yes/No). Among the statements, numbers 2, 18, 5 are designated to assess the concentration issue; numbers 1,8,15,16,24,26 for comprehension assessment; test anxiety component is incorporated in numbers 3,14,22; numbers 4,6,10,11,13,21,23 stand for organisation skill of academic information; learning issues are assigned in numbers 7,19,27; number s9 and 28 represent computer skill; and numbers 12,17,20,25 Denote the ability of taking notes properly.

Students were instructed to respond “Yes” or “No” against each of the statement. Among all 28 statements, if a student agrees by marking “Yes” in response to 10 or more statements, then he/she has some shortcomings in any of above-mentioned attributes identified. Among the seven mentioned factors identified by the tool, Lack in individual category was assessed by calculating “YES” response in each set of statements mentioned above.

MS Excel 2010 software was used for tabulation, scoring, and statistically analysing the data. Frequencies of the shortcoming of different attributes were estimated first through percentage distribution and descriptive statistics. Chi-square statistics applied between the two groups of students as mentioned above and the statistical significance of male and female students were noted. An attempt was made to find out the determining factor of performance in terms of lacking in different sections identified among the Honours and Non-honours groups using Chi-square statistics. All the statistical analyses were carried out in MS Excel software 2010 version.

## Results

**Figure 1:**
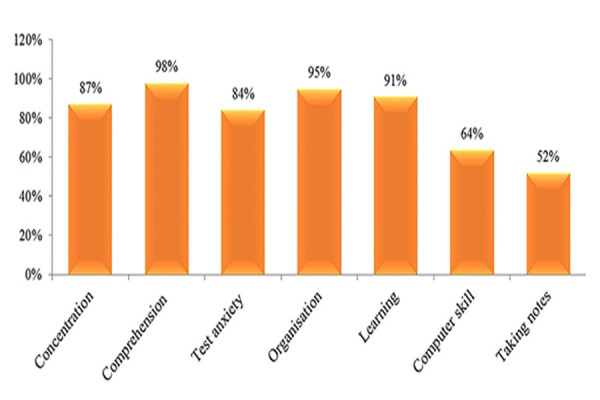
Distribution of lacking among all students (n=196)

Out of 215 students in total, 15 could not take part in this study as 9 students were absent in the class during the data collection days, and 6 of them were not willing to take part in the study. Thus, the response rate was 93% in the study. Four questionnaires were incomplete, so they were excluded. The total valid responses were 196 in number. Among them, 142 students were males (72.4%) and 54 females (27.6%). As to age distribution, the majority were in the age of 19 years (49.3%) followed by 20 years (37.2%), 21 years (13.8%), 18 years (4.6%). Among the study population (n=196), 171 respondents showed lack of concentration, whereas 192 students cannot comprehend properly. 165 out of all students were found to be suffering from test anxiety. Only 10 respondents had proper knowledge about organisation skill. 178 students had notable deficiency in learning. 125 students were deficient in computer skills. Only 94 students could take notes properly. Therefore, among the students, the maximum deficiency was seen in comprehension (98%) followed by organisational skill (95%) in the study and then l in learning (Figure 1). 

**Figure 2:**
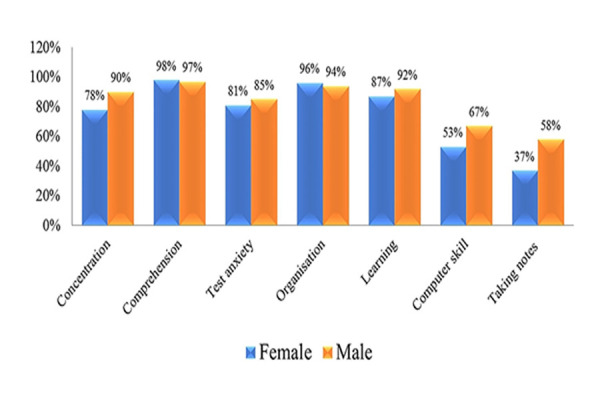
Distribution of lacking among male and female student groups (n=196)

Regarding the sex distribution of deficiency among males and females, we found comprehension and organisation skill to be almost comparable with each other (≤2% difference), but in other five attributes male students were more lagging behind (4% to 21% less) (Figure 2) than their female batch mates. 

This difference between male and female respondents was found statistically significant (p< 0.05) in concentration (p=0.008) and taking notes (p=0.009) (Table 1). 

**Table 1:** Comparison between male and female students

Attributes*	Male		Female		p value
	Lack	No lack	Lack	No lack	
Concentration	128	14	42	12	**0.008**
Comprehension	138	4	53	1	0.900
Test anxiety	121	21	44	10	0.522
Organization	134	8	52	2	0.583
Learning	131	11	47	7	0.258
Computer skill	95	47	29	25	0.070
Taking notes	82	60	20	34	**0.009**

* Seven factors, identified by Ron Fry questionnaire, which affects students’ performance.

**Figure 3:**
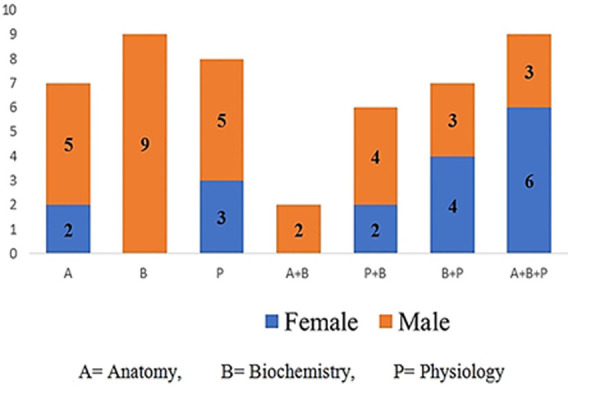
Distribution of Honours marks holders according to gender and subjects (n=48)

A student getting 75% or more marks in a particular subject is called securing ‘honours’ mark (kind of distinction in medical education in India) in the particular subject. Only 48 (24%) students secured honours, irrespective of gender and subject (Figure 3). 

**Figure 4:**
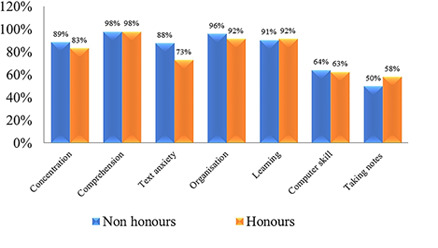
Distribution of lacking among Honours and Non-honours candidates (n=196)

Regarding difference in deficiency between honours and non-honours groups of students, we found comprehension and learning to be almost comparable (≤1%), whereas in the case of taking notes non-honours candidates were much proficient than honours candidates (Figure 4).

We found quite conclusively that test anxiety was statistically significant (p=0.013) to play a vital role as a determinant (Table 2) in division of medical students into honours candidate securing good marks and non-honours ones not having that much good results.

**Table 2:** Statistical significance of difference in lacking among Honours and Non-Honours candidates (n=196)

Attributes*	P value	Statistical significance
Concentration	0.349	False
Comprehension	0.980	False
Test anxiety	**0.013**	**True**
Organization	0.247	False
Learning	0.814	False
Computer skill	0.832	False
Taking notes	0.315	False

## Discussion

As per new Attitude, Ethics and Communication (AETCOM) module in India under Competency Based Medical Education (CBME) guideline, every Indian medical graduate (IMG) must have 5 competencies, i.e. Professional, Clinician, Lifelong learner, Leadership, and Communicator ( 6 ) whereas the Canada Physician Competency Framework mentioned three other additional qualities as collaborator, health advocate, scholar ( 7 ). Many a times, despite having good IQ even deserving students are unable to acquire the standard result ( 8 ). Starting from data acquisition followed by processing, storing, retention, transfer and recall is the learning process which, if taken care stepwise and properly, leads to a good learning outcome. The present study emphasised the assessment of the students to identify the areas of improvement, taking into consideration such a multifactorial combination of attributes which is unique of its kind and has not been considered comprehensively in totality before. Singh &amp; Kamra identified male gender, low premedical academic performance records, Internet surfing, other than for academic purpose, as negative factors for academic achievement in medical undergraduate students ( 9 ). Physical fitness, refractive error, use of smartphone and Internet, and sleeping hours before exam are some other factors ( 10 ). As revealed in a Saudi Arabian study, rather than spending time out and in social events, a social networking from smartphone durations (≤2 hours per day) play negative role in medical students’ performance ( 11 ). Lifestyle, family and relationship issues are notably mentioned ( 12 , 13 ). Moreover, daily life activities, greater academic responsibility, excessive pressure and study loads, and stressful academic and residential environment have been identified as negative factors by Thai researchers ( 14 - 16 ). Intrinsic factors include style or pattern of question paper in the examination, inappropriate sequence of questions, and teacher related factors; extrinsic factors include learning environment like temperature, suffocation in classroom, lack of discipline, noise in examination hall. Further, personal factors include selection of questions, family problems, socioeconomic status, tension, and over-confidence. Miscellaneous factors include exam phobia or test anxiety or performance anxiety ( 17 - 19 ). The satisfaction and confidence level of the studies are reflected in the exam. Concentration, comprehension and motivation to study influence the learning ability in other ways as big determinant factors in learning outcome which have been considered and measured in this study ( 20 ). Theory of constructivism says, learners construct their own knowledge based on what they already know, and they make judgement when and how to modify the knowledge because learning is an active process rather than a passive one ( 3 ). Learning is a unique style to develop improved skill of the brain through conscious practice. Taking notes properly in an organized way, group study, consulting journals, books and study materials, enough sleep, and exercise supports improve the learning process ( 21 ). Every student should understand what to learn, what to remember, and how to represent it in examinations. For solution of these problems, the university of New England proposes that interest in the subject or activity, motivation to finish a task comprehensively and successfully, level of relaxation or enjoyment, level of distraction from the environment and that from one’s own thoughts are important, and that student counselling is necessary ( 22 ). For better study or result, the University of Manitoba s every learner should study in his best time at a place of minimal distraction with a planned schedule, while having active learning strategies, keeping aside all distractions and taking breaks in between and subdivide a vast topic ( 23 ). However, every student has to deal mostly with his/her distractions, for which he/she must be accustomed to dealing with indecision, daydreaming, personal problems, botheration by a course, and also to set reminder list and realistic goal. 

A questionnaire study in Iran among 400 students (Male: Female= 1: 1) concluded that when students get the classes as diverse and challenging with assessments and tasks, they choose mastery goals, i.e. more useful and more self-regulated learning strategies. Safe environment, independence, and sense of responsibility can be used as proper learning strategies ( 24 ). In the present study, the issue of lack of concentration and comprehension was addressed which can be tackled further in a sensitive way for delivering better learning environment. Another questionnaire study conducted on 450 students in Iran revealed that the type of subject, gender, interest, and employment status were significantly related to academic performance which is quite comparable to the results of the present study ( 25 ).

Few researchers have studied the process of concentration, giving due importance to fixing of close, undivided attention and ability to direct one's thinking. Meditation was also referred as a means for improving concentration, featuring as family of self-regulation practices focusing on attention and awareness in order to bring mental processes under greater voluntary control and fostering general mental well-being and self-inducement of a mode of consciousness development ( 26 ).

Another study in Pakistan considered ten major constructs and mentioned that students were dissatisfied with many core services and facilities like teaching, administrative support, library, labs, accommodation, medical services, and sports, while satisfaction has been reported only in three augmented areas like transportation, classroom and prayer facilities ( 27 ).

As the medical course curriculum is complex and tough from the beginning, it becomes challenging to obtain good scores, which is the reason why we categorised our present study sample into honours (to assume as so called ‘good’ student) and non-honours groups to identify the gaps that make a difference in performance. The significant shortcoming in the ability of taking notes in the honours candidates can be explained as they perhaps improvise scientifically and trust in their memory and intelligence. The sex variation has also been considered to delve deep into the deficiencies. In the present AETCOM module in India, the issue of personal counselling has already been duly addressed and emphasized to relieve students from their deficiencies and ultimately contribute to a good result ( 6 ).

The present study has some limitations as there are numerous factors which affect the students’ performance and learning which fell beyond the scope of this study. Individual subject wise variation could not be considered, and only the 2nd year undergraduate students from a single institute were studied. Further extensive research covering these limitations may yield more insightful result for improvement of the situation.

## Conclusion

This study is unique in that it is the first of its kind exclusively on medical students considering exclusively seven essential affecting factors and it can be used to avail great help for implementing competency based medical education through AETCOM module in an effective way. We strongly recommend implementation of ‘teacher guardianship’ where a group of students can be taken care of personally, both academically, and emotionally using extracurricular activities, particularly by a teacher beyond the class hours, i.e. individualised care. We also recommend this tool to be used and compared after each year’s university exam to follow up the students’ improvement. Students’ friendly approach in the class and psychological counselling is also important. Thus, a narrower gap between the teacher’s intension and learner’s interpretation can be achieved for better result if teachers act more like a learning facilitator than mere an information provider. 
